# Facile Synthesis and Preferred Conformation Analysis of Cyclododeceno[b]indene

**DOI:** 10.3390/molecules15020699

**Published:** 2010-02-01

**Authors:** Chunyan Zhang, Shengyan Gong, Li Zhang, Daoquan Wang, Mingan Wang

**Affiliations:** Department of Applied Chemistry, China Agricultural University, Beijing 100193, China

**Keywords:** α-benzylcyclododecanone, cyclododeceno[b]indene, synthesis, conformational analysis

## Abstract

Using methanesulfonic acid as a catalyst, a series of cyclododeceno[b]indene derivatives were synthesized by the cyclization of α-benzylcyclododecanones, which were prepared by the reactions of cyclododecanones with a variety of substituted benzyl chlorides or bromides using NaH as a base. Their structures were confirmed by mp, IR spectra, ^1^H-NMR, ^13^C-NMR, MS, and x-ray diffraction. The preferred conformations were analyzed by crystal structure, ^1^H-NMR and quantum chemistry calculations, and compared with the x-ray diffraction structure of 2,3,5,6-bis(*ortho*-1,10-decylidene)dihydropyrazine. The results showed that the cyclododecene moiety adopted a preferred [1ene2333] conformation, and the substituted groups at aromatic ring had no significant influence on the conformation.

## 1. Introduction

Benzocyclopenta-1,3-diene (indene) is a compound with an 8π-electron system in a planar conformation that makes it easy to lose one proton and form a 10π-electron aromatic system, and therefore plays an important role in Organic Chemistry. Indene derivatives are widely used as useful intermediates in the development of drug molecules, pesticides, and functional materials [1,2]. They have also been used as metal ligands of Ziegler-Natta type catalysts, and these complexes are highly stable and have excellent catalytic efficacy in the polymerization of ethylene and propylene [3,4]. Cyclohexeno[b]indene in particular has been used as a Ziegler-Natta catalyst metal ligand [5]. In another aspect, bi- and tricyclic compounds with bridge double bonds are very useful starting reagents for the synthesis of macrocyclic compounds such as the fragrant macrocyclic lactone pentadecanolide and related compounds [6,7]. Synthesis of indene derivatives containing cyclic olefins ranging from 5 to 12-membered rings have been reported, with the exception of the corresponding 11-membered ring compounds. As for cyclododeceno[b]indene, it has been reported by Parham and coworkers [8] that this compound can be synthesized in five steps using cyclododecanone and a Grignard reagent as starting materials. However the reaction conditions are difficult to control, the intermediates are hard to purify, and the overall yield is low. Zakharkin and coworkers [9] reported a convenient method in which α-benzylcyclododecanones were synthesized by the reaction of cyclododecanone with benzyl chloride under phase transfer catalysis, followed by cyclization of α-benzylcyclododecanone using polyphosphoric acid as the catalyst. Recently, Miyamoto and coworker [10] reported a Rh(I)-catalyzed reaction of 2-(chloromethyl)phenylboronic acid and cyclododecyne leading to cyclododeceno[b]indene in 36%–54% yields, but cyclododecyne is not readily available. 

**Scheme 1:**
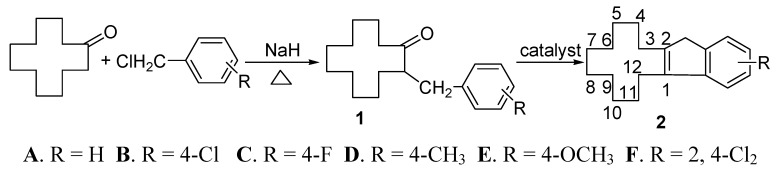
The synthetic route of cyclododeceno[b]indenederivatives.

So far, cyclododeceno[b]indene derivatives have not been evaluated for their catalytic efficacy as metal ligands of Ziegler-Natta type catalysts. Moreover, it is not clear whether the conformation of the cyclododecene ring in cyclododeceno[b]indene molecules has any significant impact on the catalytic efficacy of these compounds. In order to resolve these issues and extend our research on the stereochemistry of 12-membered ring systems [11,12,13,14,15], we have synthesized a series of cyclododeceno[b]indene derivatives using methanesulfonic acid as catalyst and carried out the conformational analysis of these compounds. The synthetic route is shown in Scheme 1. The evaluation of the catalytic efficacy of these compounds are still in progress in our laboratory. 

## 2. Results and Discussion

**Scheme 2:**
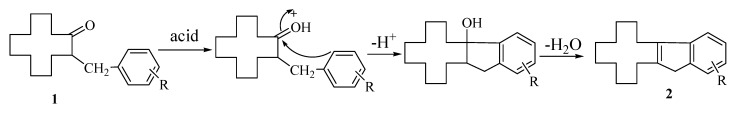
The cyclization mechanism of the title compounds.

In the synthesis of the title compounds, we found that the cyclization of intermediates **1** did not take place after several days using the Zakharkin method when *n*-hexane was used as solvent and PPA or phosphoric acid as the catalysts [9]. We next used the more acidic *p*-methylbenzenesulfonic acid as the catalyst, which enabled the reaction of α-benzylcyclododecanone (**1A**) to produce compound **2A** in 30% yield, but *para*-fluoro- or *para*-chloro-α-benzylcyclododecanones **1B** and **1C** did not react under similar conditions. This may be due to the heterogenous nature of these reactions because *p*-methyl-benzenesulfonic acid remained in a solid state in the reaction mixtures, which resulted in poor catalytic efficacy of the reagent. Then we used anhydrous aluminum chloride as the catalyst as in [5], where Thomas reported the synthesis of cyclohexeno[b]indene by the cyclization reaction of α-benzylcyclo-hexanone in the presence of anhydrous aluminum chloride. The results showed that in hexane the products were complex and difficult to purify when **1A**–**4A** were used as the starting materials. Considering the characteristics of the dehydration after the electrophilic cyclization, as indicated in Scheme 2, we tried methanesulfonic acid as the catalyst in an amount that was 2–3 times the amount of α-benzylcyclododecanone used. As a result, we found that the reaction time was shortened, the products were relatively easier to isolate, and the yields were in the 5–16% range for **2A**, **2C**, **2D **and **2E. **Compound **2B **was an exception**. **Encouraged by these results, we added a 10-fold excess of methanesulfonic acid without changing the other conditions, and were able to achieve yields of 35–90% for **2A**, **2B**, **2C**, **2D **and **2E**,respectively. Compound **1F** still did not afford **2F**. Comparing these results with the reaction yields and conditions used for α-benzylcyclohexanone [5], we found that it is more difficult to obtain the cyclization products of α-benzylcyclododecanone, which may be attributed to the conformational difference between the six-membered and twelve-membered ring ketones involved [16].

**Figure 1:**
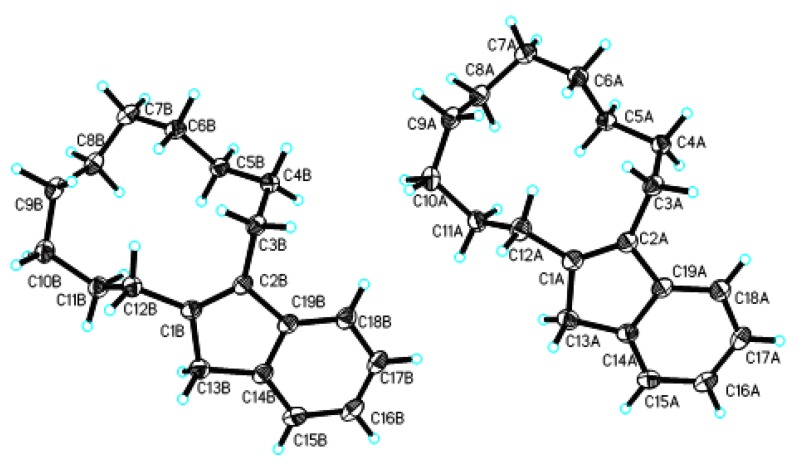
The crystal structure of compound **2A**.

The preferred conformation of *cis-*cyclododecene and its derivatives is [1ene2333], as discussed by several research groups [17,18,19,20], but research on the preferred conformation of *trans-*cyclododecene is rather minimal [21] and a fundamental conclusion has not yet been reached. The title compounds **2B**–**2D** are colorless crystals, but our attempts at growing a suitable single crystal in *n*-hexane and cyclohexane were not successful, while **2A** was successfully crystallized from *n*-hexane and subjected to x-ray diffraction analysis. The results (Figure 1) show that a *cis*-configuration and the preferred [1ene2333] conformation for the *cis*-cyclododecene moiety are present in the crystals of **2A**, and the benzocyclopenta-1,3-diene moiety adopts a planar structure. The preferred [1ene2333] conformation of the *cis*-cyclododecene moiety was consistent with that seen in the crystals of other similar molecules like 15-phenylbicyclo-[10,3,0]pentadec-1(12)-en-13-one [19], 1-methoxycarbonyloxyl-2-phenoxy- carbonyl-1,2-cyclododecene, 1-phenoxycarbonyloxyl-2-methoxycarbonyl-1,2-cyclododecene [11], and 2,3,5,6-bis(*ortho*-1,10-decylidene)dihydropyrazine [15].

In the [1ene2333] conformation, the two protons of the 3-CH_2_ and 12-CH_2_ occupy side-*exo* and side-*endo*-positions, respectively, and therefore they should display different chemical shifts in the ^1^H- NMR spectrum. However, in the ^1^H-NMR spectra of **2A**–**2E**, the two protons on each side of the asymmetric 1,2-disubstituted cyclododecene appeared as equivalent protons which are coupled with their adjacent protons to give triplet signals with 7.0 Hz coupling constants. These results are consistent with the ^1^H-NMR characteristics of asymmetric 1,2-disubstituted cyclododecene reported in our previous paper [11,15], and show that **2** may adopt two different [1ene2333] conformations like other asymmetric 1,2-disubstituted cyclododecenes, which coexist in a dynamic equilibrium in solution [11,19,20]. The ^1^H-NMR spectra in solution are the averaged results of these two different [1ene2333] conformations. Compound **2A **adopted one of the two conformations in its crystal form.

**Figure 2:**
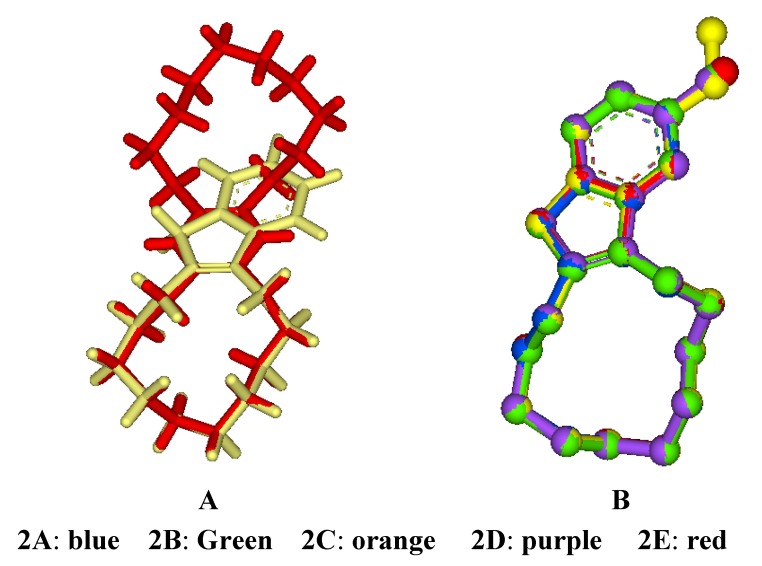
Superimposed diagrams of **2A**–**2E **and 2,3,5,6-bis(*ortho*-1,10-decylidene)-dihydropyrazine.

In order to validate our conclusions, the quantum chemistry method was used to analyze the conformation of the title compounds. The conformational optimization was performed for **2A**–**2E **using the Gaussian 03 software and the results compared with the x-ray diffraction structures of **2A** and 2,3,5,6-bis(*ortho*-1,10-decylidene)dihydropyrazine [15]. Vibrational frequencies were computed at the same level, and the positive values of the first frequency for the title compounds showed the optimized conformations were the local energy minimum conformations and located the stationary points. Using **2A** as example, its optimized structure was completely consistent with the obtained x-ray structure and it also showed the same [1ene2333] conformation of 2,3,5,6-bis(*ortho*-1,10-decylidene)dihydro-pyrazine, as seen in the superimposed diagrams in Figure 2(A). Similarly, we analyzed the optimized structures of **2B**–**2E **and superimposed them with that of **2A** as seen in Figure 2(B). They all matched pretty well, and the conformational differences between the *cis*-cyclododecene and the benzocyclopenta-1,3-diene moieties were insignificant. These results implied that the cyclododecene moiety in the optimized structures of **2A**–**2E **adopts a [1ene2333] conformation, and the indene ring and its substituents have no significant influence on the conformation of the cyclododecene moiety.

We also compared the torsion angles of **2A**–**2E** (Table 1), and found that the changes of all twelve torsion angles of **2A**–**2E **are in the range of 0-1°, indicating that the structural differences between the five compounds are minimal. Further, we compared the torsion angles of **2A**–**2E** with those of 2,3,5,6-bis(*ortho*-1,10-decylidene)dihydropyrazine, the changes of all twelve torsion angels are in the range of 0-9.1°, especially the angels C1-C2-C3-C4, C2-C3-C4-C5 and C3-C4-C5-C6 are 9.1, 6.7 and 3.8, respectively. This may be due to the various influences of different rings connecting cyclododecene and the relative distances near the double bond of cyclododecene. The larger the relative distance is, the smaller the torsion angel changes. The other torsion angels showed only 0-3° difference. Again, this comparison indicated that the *cis*-cyclododecene moiety in **2A**–**2E** and 2,3,5,6-bis (ortho-1, 10-decylidene) dihydropyrazine take the same [1ene2333] preferred conformation.

**Table 1:** The torsion angles of **2A**-**2E **and 2,3,5,6-bis(*ortho*-1,10-decylidene)dihydropyrazine.

Angle	Torsion angle (°)					
	2A	2B	2C	2D	2E	Ref. [13]
C1-C2-C3-C4	-117.0(-118.1^a^)	-117.0	-117.1	-117.0	-117.2	-126.1
C2-C3-C4-C5	162.0(166.6)	162.7	162.5	162.8	162.6	168.7
C3-C4-C5-C6	-65.2(-65.0)	-65.0	-64.9	-65.0	-64.8	-61.4
C4-C5-C6-C7	-64.5(-64.5)	-64.2	-64.3	-64.2	-64.2	-63.3
C5-C6-C7-C8	150.8(147.2)	150.1	150.3	150.0	150.2	148.9
C6-C7-C8-C9	-65.1(-64.6)	-65.3	-65.3	-65.3	-65.3	-65.1
C7-C8-C9-C10	-62.7(-62.2)	-63.1	-63.3	-63.0	-63.4	-61.5
C8-C9-C10-C11	175.6(178.9)	176.0	175.7	176.1	175.8	177.8
C9-C10-C11-C12	-74.3(-73.1)	-73.7	-73.4	-73.7	-73.3	-76.3
C10-C11-C12-C1	-73.0(-71.2)	-73.0	-73.1	-72.9	-73.0	-74.2
C11-C12-C1-C2	105.4(100.8)	105.0	105.4	104.8	105.2	102.4
C12-C1-C2-C3	-1.5(-2.4)	-1.8	-1.8	-1.7	-1.8	-0.6

**^a^**These data are from the x-ray diffraction crystal structure of **2A**.

Thomas [5] found that metallocenes containing indene were highly stable and efficient Ziegler-Natta type catalysts for the polymerization of ethylene and propylene, but that lower polymer molecular weights were obtained with the systems, which were attributed to the more indenyl-like character of the systems or to the less rigid geometry of cyclohexene ring in the complexes. Based on this observation, the relatively rigid [1ene2333] conformation of the cyclododecene ring in the cyclododeceno[b]indene derivatives may be helpful to improve polymer molecular weights in the polymerization of ethylene and propylene when using cyclododeceno[b]indene derivatives as Ziegler-Natta type catalyst ligands. This is the subject of further studies being performed by our group. 

## 3. Experimental

### 3.1. General

Melting points were measured on a Yanagimoto NFG CO apparatus and are uncorrected; IR spectra were determined on an IR-450 instrument; ^1^H-NMR and ^13^C-NMR spectra were recorded on a Brüker DPX 300 NMR spectrometer with CDCl_3_ as solvent and TMS as internal standard. APCI-MS was recorded with an Agilent LCQ LC-MSD ion-trap mass spectrometer. Cyclododecanone (99.8%) was purchased from Acros Organics; *p*-chlorobenzyl chloride (99%) and *p*-fluorobenzyl chloride (99%) were from Johnson Matthey; *p*-methylbenzyl chloride (99%) was from Alfa Aesar; *o*,*p*-dichlorobenzyl chloride (98%) was from Avocado; *p*-methoxybenzyl alcohol (98%) was from Merck；NaH (80%) was from Beijing Chemical Reagent Co.；PPA (80%, P_2_O_5_), anhydrous aluminum chloride (99%) and methanesulfonic acid (98.5-101%) were from Beijing Yili Chemicals Co.; *p*‑methylbenzenesulfonic acid (98%) was from Shanghai Reagent factory; the solvents were analytical grade and treated with sodium and benzophenone before usage.

### 3.2. Crystallographic Data

CCDC 759652 contains the supplementary crystallographic data for this paper. These data can be obtained free of charge via www.ccdc.cam.ac.ck/conts/retrieving.html (or from CCDC, 12 Union Road, Cambridge CB2 1EZ, UK. Fax: +44 1223 336033; E-Mail: deposit@ccdc.cam.ac.uk).

### 3.3. General Synthetic Method for Intermediates ***1A–1F***

Compounds **1A**–**1F** were synthesized in 57–80% yields according to the procedure reported in [12,22]. Among them, *p*-methoxybenzyl bromide was prepared using *p*-methoxybenzyl alcohol and hydrogen bromide [23], and utilized in the next reaction without further purification. The mp**, **IR**, **^ 1^H- and ^13^C-NMRof **1A** were consistent with the data in the literature [12].

*α-4-Chlorobenzylcyclododecanone* (**1B**): Colorless crystal, yield 61%, mp: 80–82 °C. IR υ: 2930, 1710, 1590, 1490, 1430, 1400, 1355, 1105, 1020, 1000, 855, 810, 720, 700 cm^-1^. ^1^H-NMR δ: 7.20-7.26 (m, 2H), 7.06-7.10 (m, 2H), 2.85-2.94 (m, 2H), 2.49-2.62 (m, 2H), 2.16-2.25 (m, 1H), 1.54-1.76 (m, 4H), 1.29-1.25 (m, 14H). ^13^C-NMR δ: 213.62, 138.41, 131.91, 130.16, 128.48, 53.19, 38.46, 36.50, 29.31, 26.09, 25.76, 23.84, 23.67, 22.33, 22.24, 21.83.

*α-4-Flurobenzylcyclododecanone* (**1C**): Colorless crystal, yield 60%, mp: 68–69 °C. IR υ: 2920, 1715, 1595, 1490, 1435, 1400, 1355, 1105, 1020, 1000, 850, 810, 720, 705 cm^-1^. ^1^H-NMR δ: 7.07-7.13 (m, 2H), 6.91-6.98 (m, 2H), 2.85-2.92 (m, 2H), 2.47-2.65 (m, 2H), 2.16-2.26 (m, 1H), 1.54-1.75 (m, 4H), 1.29-1.26 (m, 14H). ^13^C- NMR δ: 213.88, 161.39 (^1^*J*_FC_ = 240.0 Hz), 135.53 (^4^*J*_FC_ = 3.1 Hz), 130.19 (^3^*J*_FC_ = 7.8 Hz), 115.13 (^2^*J*_FC_ = 21.1 Hz), 53.38, 38.52, 36.47, 29.35, 25.73, 24.10, 23.90, 23.75, 22.43, 22.24, 21.85.

*α-4-Methylbenzylcyclododecanone* (**1D**): Colorless crystal, yield 57%, mp: 68–70 °C. IR υ: 2925, 1718, 1605, 1495, 1430, 1405, 1360, 1100, 1020, 1010, 855, 805, 720 cm^-1^. ^1^H-NMR δ: 7.09-7.02 (m, 4H), 2.94-2.83 (m, 2H), 2.63-2.44 (m, 2H), 2.30-2.22 (m, 4H), 1.71-1.57 (m, 4H), 1.28-1.27 (m, 14H). ^13^C-NMR δ: 214.35, 136.74, 135.62, 129.08, 128.71, 53.39, 38.51, 37.18, 29.44, 25.74, 24.20, 24.07, 23.96, 22.72, 22.33, 21.95, 20.98.

*α-4-Methoxybenzylcyclododecanone* (**1E**): Colorless crystal, yield 78%, mp: 75–76 °C. IR υ: 2920, 1700, 1590, 1500, 1450, 1430, 1250, 1170, 1100, 1050, 950, 800, 720, 690 cm^-1^. ^1^H-NMR δ: 7.09-7.04 (m, 2H), 6.83-6.78 (m, 2H), 3.78 (s, 3H), 2.91-2.80 (m, 2H), 2.62-2.57 (m, 1H), 2.52-2.42 (m, 1H), 2.29-2.19 (m, 1H), 1.71-1.28 (m, 4H), 1.27(m, 14H). ^13^C-NMR δ: 214.47, 158.00, 131.86, 129.74, 113.82, 55.20, 53.50, 38.60, 36.78, 29.45, 26.10, 25.69, 24.18, 24.06, 23.96, 22.71, 22.29, 21.94.

*α-2,4-Dichlorobenzylcyclododecanone* (**1F**): Colorless crystal, yield 76%, mp: 94–95 °C. IR υ: 2920, 1700, 1590, 1470, 1430, 1400, 1350, 1100, 1020, 1000, 850, 800, 720, 700 cm^-1^. ^1^H-NMR δ: 7.36-7.33 (m, 1H), 7.17-6.94 (m, 2H), 3.12-2.99 (m, 2H), 2.72-2.60 (m, 2H), 2.20-2.10 (m, 1H), 1.81-1.53 (m, 2H), 1.29-1.22 (m, 16H). ^13^C-NMR δ: 213.48, 136.31, 132.69, 132.44, 129.35, 126.98, 126.88, 50.44, 38.96, 34.32, 29.40, 26.01, 25.62, 24.16, 24.01, 23.86, 22.35, 22.33, 21.94.

### 3.4. General Synthetic Method for the Title Compounds ***2A–2E***

Taking **2D **as an example: anhydrous *n*-hexane (30 mL) and 1.3 mL (0.02 mol) methylsulfonic acid were added to a 100 mL three-necked bottle, then 0.6 g (0.002 mol) α-(4-methylbenzyl) cyclododecanone was added into the bottle under stirring and heated to keep reflux. After the reaction was completed (three days after TLC check), water (30 mL) was added to the mixture. The mixture was extracted three times with n-hexane (3 × 30 mL), and the combined organic layer was washed with saturated NaCl solution, dried with anhydrous MgSO_4_, filtered and evaporated under reduced pressure to give an pale yellow oil. The oil was chromatographed on a silica gel column and washed with redistilled petroleum ether. 0.5 g colorless crystal was afforded in 90% yield. Similarly, **2A**–**2E **was synthesized successfully, but **2F** was an exception.

*Cyclododeceno[b]indene* (**2A**): Colorless crystals, mp 53–55 °C, 50% yield. IR υ: 2950, 1620, 1595, 1490, 1435, 1400, 1350, 1105 cm^-1^. ^1^H-NMR δ: 7.39-7.08 (m, 4H), 3.28 (s, 2H), 2.58 (t, *J* = 7.0 Hz, 2H), 2.47 (t, *J* = 7.2 Hz, 2H), 1.76-1.65 (m, 4H), 1.52-1.24 (m, 12H). ^13^C-NMR δ: 146.73, 143.79, 142.87, 136.93, 125.92, 123.47, 123.21, 118.85, 39.96, 27.17, 26.04, 25.30, 25.15, 25.08, 24.92, 23.69, 22.80, 22.33, 22.00. NMR data were consistent with the data in the literature [6,8]. APCI-MS: 255 [M+H]^+^. 

*3-Chlorocyclododeceno[b]indene* (**2B**): Colorless crystals, mp 78–80 °C, 35% yield. IR υ: 2960, 1625, 1590, 1495, 1430, 1405, 1350, 1110 cm^-1^. ^1^H-NMR δ: 7.27-7.23 (m, 2H), 7.08-7.05 (dd, *J* = 7.8, 2.0 Hz , 1H), 3.25 (s, 2H), 2.52 (t, *J* = 7.0 Hz, 2H), 2.47 (t, *J* = 7.2 Hz 2H), 1.74-1.64 (m, 4H), 1.50-1.22 (m, 12H). ^13^C-NMR δ: 148.61, 145.88, 141.03, 136.57, 132.06, 123.99, 123.29, 119.11, 39.59, 27.14, 25.95, 25.42, 25.14, 25.12, 24.94, 23.75, 22.79, 22.28, 22.05. APCI-MS: 289 [M+H]^+^.

*3-Flurocyclododeceno[b]indene* (**2C**): Colorless crystals, mp 50–52 °C, 46% yield. IR υ: 2955, 1620, 1590, 1495, 1440, 1410, 1355, 1105 cm^-1^. ^1^H-NMR δ: 7.28-7.24 (m, 1H), 6.98-6.94 (m, 1H), 6.81-6.75 (m, 1H), 3.24 (s, 2H), 2.54 (t, *J* = 7.0 Hz, 2H), 2.47 (t, *J* = 7.2 Hz, 2H), 1.74-1.64 (m, 4H), 1.51-1.23 (m, 12H). ^13^C-NMR δ: 162.45 (^1^J_FC_ = 240.9 Hz), 148.75 (^3^J_FC_ = 8.4 Hz), 146.38, 137.95 (^4^J_FH_ = 2.5 Hz), 136.73 (^4^J_FH_ = 2.9 Hz), 123.66 (^3^J_FH_ = 9.1 Hz), 109.83 (^2^*J*_FH_ = 22.9 Hz), 106.05 (^2^*J*_FH_ = 22.9 Hz), 39.37, 27.14, 25.91, 25.46, 25.14, 25.10, 24.91, 23.72, 22.78, 22.31, 22.02. APCI-MS: 273 [M+H]^+^.

*3-Methylcyclododeceno[b]indene* (**2D**): Colorless crystals, mp 50–52 °C, 90% yield. IR υ: 2950, 1624, 1595, 1490, 1445, 1405, 1350, 1110 cm^-1^. ^1^H-NMR δ: 7.25 (d, *J* = 7.5 Hz, 1H), 7.10 (d, *J* = 1.5 Hz, 1H), 6.92 (dd, *J* = 7.5 1.5 Hz, 1H), 3.24 (s, 2H), 2.56 (t, *J* = 6.9 Hz, 2H), 2.46 (t, *J* = 7.2 Hz 2H), 2.39(s, 3H), 1.76-1.63 (m, 4H), 1.51-1.24(m, 12H). ^13^C-NMR δ: 146.99, 144.08, 139.92, 136.85, 135.44, 124.24, 122.89, 119.67, 39.58, 27.21, 26.10, 25.38, 25.19, 25.12, 25.00, 23.78, 22.85, 22.37, 22.06, 21.58. APCI-MS: 269 [M+H]^+^.

*3-Methoxycyclododeceno[b]indene* (**2E**): Light yellow oil, 62% yield. IR υ: 2960, 1625, 1592, 1495, 1450, 1415, 1340, 1115 cm^-1^. ^1^H-NMR δ: 7.26(d, *J* = 7.8 Hz, 1H), 6.85 (d, *J* = 2.4 Hz, 1H), 6.66 (dd, *J* = 7.8,2.4 Hz, 1H), 3.83 (s, 3H), 3.23(s, 2H), 2.56 (t, *J* = 6.9 Hz, 2H), 2.46 (t, *J* = 7.2 Hz 2H), 1.73-1.44 (m, 4H), 1.39-1.24 (m, 12H). ^13^C-NMR δ: 158.85, 148.25, 145.47, 136.83, 135.02, 123.44, 108.77, 105.34, 55.55, 39.22, 27.17, 26.04, 25.47, 25.18, 25.13, 24.96, 23.73, 22.84, 22.34, 22.04. APCI-MS: 285 [M+H]^+^.

### 3.5. X-Ray Diffraction of Compound ***2A***

The crystal of compound **2A **was obtained by slow evaporation of a hexane solution. X-ray diffraction analysis: all measurements for 0.50 × 0.49 × 0.45 mm crystal were made with a Rigaku R-axis RAPID IP four circle area detector using graphite monochromatized Mo Kα (*λ* = 0.071073 nm) radiation at 73 K. Full spheres of data were collected to a 2θ limit of 25.00°. 19888 reflections were collected with 5233 unique [R(int) = 0.0366], 4929 reflections were stronger than 2σ in intensity. Space groups were determined from systematic absence and checked for higher symmetry. The structures were solved by direct methods using SHELX, and refined on *F^2^* using all data by full-matrix least-squares procedures with SHELXL-97. All non-hydrogen atoms were refined with anisotropic displacement parameters. An empirical absorption correction based on Xscans was made on all data. Hydrogen atoms were located from the difference map and were constrained to geometrical estimates. Final refinement was carried out with isotropic displacement parameters applied to hydrogen atoms. The crystal structure parameters for **2A** was: C_19_H_26_, *M_r_*** = **254.40, monoclinic, space group *P2(*1)/c, *a* = 1.4406(3), *b* = 1.6472(3), *c* = 1.2580(3) nm, *β* = 93.36(3)°, *V* = 2.9799(10) nm^3^, *Dc* = 1.134 g/cm^3^, *Z* = 8, *F*(000) = 1120, μ(Mo *Kα*) = 0.063 mm^-1^, final *R* = 0.0653, *wR* = 0.1725.

### 3.6. Conformational Optimization of Compounds ***2A–2E***

The geometry optimizations were performed by the DFT method using the B3LYP functional [24,25]. A standard valence double-zeta with polarization function 6-31G (d, p) basis set [26] was used for all kinds of atoms involved in five target molecules (**2A**–**2E**). Vibration frequencies were computed at the B3LYP/6-31G (d, p) levels to characterize stationary points. All calculations were performed using the Gaussian 03 software [27].

## 4. Conclusions

Five cyclododeceno[b]indene derivatives were easily prepared in good yields using α-benzyl- cyclododecanone as the raw material, and a 10-fold excess of methanesulfonic acid as catalyst. The preferred conformations were analysized by x-ray diffraction, ^1^H-NMR and quantum chemistry calculations, and compared with the X-ray diffraction structure of 2,3,5,6-bis(*ortho*-1,10-decylidene)-dihydropyrazine. The results have shown that the *cis*-cyclododecene moiety in the title compounds adopts a preferred [1ene2333] conformation, and that while two asymmetric [1ene2333] conformations coexist in a dynamic equilibrium in solution, only one [1ene2333] conformation is present in the crystal solid, and the substituted groups at aromatic ring do not have significant influence on the conformation of the cyclododecene moiety.
